# The Optimal Supplementation of Fermented Product Produced by *Bacillus subtilis* Strain LYS1 with High Surfactin Yield for Improving Growth Performance, Intestinal Villi Morphology, and Tibial Bone Strength in Broilers

**DOI:** 10.3390/ani14142079

**Published:** 2024-07-16

**Authors:** Yueh-Sheng Lee, Kuo-Lung Ku, Chi-Shih Chu, Kuo-Lung Chen

**Affiliations:** 1 Ph.D. Program of Agriculture Science, National Chiayi University, Chiayi 600355, Taiwan; lopqkojhog@gmail.com; 2 Department of Applied Chemistry, National Chiayi University, Chiayi 600355, Taiwan; klku@mail.ncyu.edu.tw; 3 Department of Microbiology, Immunology and Biopharmaceuticals, National Chiayi University, Chiayi 600355, Taiwan; cschu@mail.ncyu.edu.tw; 4 Department of Animal Science, National Chiayi University, Chiayi 600355, Taiwan

**Keywords:** *Bacillus subtilis* LYS1, broiler, growth performance, surfactin, tibial bone characteristic

## Abstract

**Simple Summary:**

*Bacillus subtilis* is a probiotic that can produce surfactin during fermentation. Surfactin is a lipopeptide whose antibacterial activity increases positively with fatty acid chain length, showing great potential to replace antibiotic growth promoters. *B. subtilis* LYS1 (LYS1) is selected as a strain that can produce high surfactin yields. In addition, previous research has confirmed that surfactin content and LYS1 counts in the fermented product (FP) are positively correlated with weight gain and production efficiency factor in broilers. This study shows that dietary supplementation with 1 to 2.5% LYS1 FP can improve the growth performance, development of intestinal villi, and tibial bone density/mineralization in broilers, and that 1.9% is the optimal supplementation level of LYS1 FP in the diet.

**Abstract:**

This study aimed to investigate the physiochemical characterizations of the fermented product (FP) produced by the high-yield surfactin strain *Bacillus subtilis* LYS1 (LYS1), as well as its effects on growth performance, carcass traits, intestinal morphology, tibial bone characteristics, and clinical blood biochemistry in broilers. Accordingly, the optimal supplementation of FP for improving growth performance, intestinal villi development, and tibial bone strength in broilers was elucidated using a broken-line quadratic (BLQ) ascending model. Three hundred and sixty 0-day-old Ross 308 broiler chicks, with equal numbers of both sexes, were randomly assigned to dietary supplementation of 2.5% fish meal or 0, 1, 1.5, 2, or 2.5% FP. Each treatment had six replicates, and the experimental period was 5 wk. The LYS1 count, surfactin content, and surfactin composition of the FP were 9.1 log CFU/g, 11.23 mg/g, and C_12_ to C_18_, respectively. The FP-supplemented groups improved feed intake, weight gain (WG), and production efficiency factor at 0 to 5 weeks old (*p* < 0.05) compared with the 0% group. The villus height/crypt depth (V/C) in the jejunum and ileum of the FP-supplemented groups was higher than in the 0% group (*p* < 0.05). The tibiotarsal index, Ca, and P in the tibia showed a linear effect with increased FP supplementation (*p* < 0.05). Moreover, the tibiotarsus weight/length index (TWLI) showed a quadratic effect with increased FP supplementation (*p* < 0.05). The optimal supplementation of FP for WG, V/C in the jejunum and ileum, and TWLI was 1.8, 1.9, and 1.6%, respectively. In conclusion, dietary supplementation with 1 to 2.5% LYS1 FP in broilers can improve their growth performance and the development of intestinal villi. Moreover, 1.9% is the optimal supplementation of LYS1 FP in the diet, based on the fitting results obtained with the BLQ model.

## 1. Introduction

*Bacillus subtilis* can generate endospores and has better survival ability than other probiotics during the feed process. Moreover, in terms of the practical functions of *B. subtilis* in animal guts, it has been reported that their endospores can resist low pH values, bile salts, and other digested enzymes [1,2]. *B. subtilis* is normally applied in the poultry industry as a direct-fed microbial, improving the growth performance, development of intestinal villi, and tibial health in broilers [3,4]. Surfactin, a secondary metabolite produced by *Bacillus* spp. through the non-ribosomal peptide synthetase, is an amphiphilic lipopeptide. In general, surfactin consists of fatty acid chains ranging in length from 12 to 17 carbon atoms, where C_14_ and C_15_ account for the highest proportions in its composition [5]. The antibacterial activity of surfactin increases positively with fatty acid chain length [6]. Accordingly, the diverse combinations of surfactin have a wide range of antibacterial activities, showing great potential to replace antibiotic growth promoters [7,8,9].

Recent studies have indicated that directly feeding poultry with *Bacillus* spp. fermented product (FP) or powder produced by their strains, which contains surfactin, can improve the development of intestinal villi, adjust the intestinal microflora, and promote the growth of broilers [10,11,12]. Cheng et al. [11] reported that supplementation with 0.3% *B. subtilis* NIU068 FP (surfactin content: 0.3 mg/kg feed) could improve growth performance, intestinal villus height, and crypt depth in broilers. Chen and Yu [13] found that supplementation with 0.3% *B. licheniformis* FP (surfactin content: 14.1 mg/kg feed) could improve the ADG at 1 to 35 d old and adjusted the abundance of the genus *Lactobacillus* in feces, which was positively correlated with ADG. Nevertheless, few studies have investigated the effects of surfactin content and its composition in FP on growth and physiology in broilers simultaneously.

*B. subtilis* LYS1 (LYS1) is a selected strain known for its ability to produce high yields of surfactin, as previously reported by the authors [4]. The surfactin content in FP produced by LYS1 through solid-state fermentation for 2 days exceeded 10 mg/g and included surfactin compositions from C_12_ to C_17_, with a notable 6.96% proportion of long carbon chains (C_16_ and C_17_), which was higher compared to other FPs produced by selected *Bacillus* strains. Furthermore, Lee et al. [4] confirmed that surfactin content and *Bacillus*-like counts in FP were positively correlated with weight gain and production efficiency factor. Consequently, supplementation with 2.5% LYS1 FP improved the growth performance and intestinal villi development in broilers. The above results imply that the surfactin composition and content in the FP have important effects on the growth performance of broilers; thus, analyzing the surfactin characteristics of FP is one of the objectives of this study. The growth performance of broilers is inseparable from their intestinal absorption capacity and bone health. Good intestinal villus physiology can help in effectively utilizing nutrients for body growth, while strong tibial bones can prevent leg diseases to support the rapid growth of broilers [3,14]. Guo et al. [15] indicated that supplementation with 0.5% *B*. *subtilis* PB6 could improve the overall average daily gain and tibial characteristics. However, the optimal supplementation of LYS1 FP for improving growth performance, intestinal villi morphology, and tibial bone strength in broilers still needs to be clarified.

This study aimed to investigate the physiochemical characterizations and surfactin characteristics of FP with high surfactin content produced by LYS1, along with its effects on growth performance, carcass traits, intestinal morphology, tibial bone characteristics, and clinical blood biochemistry in broilers. Finally, the optimal supplementation of surfactin content and LYS1 counts in FP on improving growth performance, intestinal villi development, and tibial bone strength in broilers was elucidated using a broken-line quadratic (BLQ) ascending model.

## 2. Materials and Methods

### 2.1. FP Preparation

The high-yield surfactin strain LYS1, as identified by Lee et al. [4], was used as the fermented strain for FP preparation. Soybean meal was sterilized at 121 °C at 1.21 kg/cm^2^ for 30 min, then cooled down to 45 °C. The LYS1 counts at a concentration of 10^6^ CFU/g of substrate were premixed and inoculated. Subsequently, the water content of the substrate was adjusted to 55% in order to facilitate aerobic fermentation at 37 °C for two days. The FP was dried in an oven at 55 °C until the water content was below 12% and was then ground to produce FP powder.

### 2.2. Animal Management and Experimental Design

Three hundred and sixty 0-day-old Ross 308 broiler chicks with initial body weight (BW) of 43 ± 1 g and equal numbers of both sexes, under a randomized complete block design, were randomly assigned to dietary supplementation with 2.5% fish meal or 0, 1, 1.5, 2, or 2.5% FP. Each treatment had six replicates (with 10 broiler chicks per pen). The experimental period was 5 wk. Feed and water were provided ad libitum throughout the experimental period. Diet formulation referred to the recommendation of Ross broilers [16], and the management of the broilers followed the *Ross Broiler Management Manual* [17]. The feed composition analysis is presented in Table 1. The Institutional Animal Care and Use Committee of National Chiayi University (IACUC, protocol number 110018) approved all of the procedures used in this experiment.

### 2.3. Measurements and Analysis

#### 2.3.1. The Physiochemical Characterizations of FP

The FP underwent serial dilution in 0.9% NaCl solution and was then placed on tryptic soy agar (HIMEDIA^®^, Mumbai, MH, India) at 37 °C for 24 h. The counts of the LYS1 colonies were quantified as colony-forming units per gram (CFU/g). The pH of the FP sample was determined using a pH meter (PB-10, Sartorius^®^, Göttingen, Germany), while the water content was assessed with an infrared moisture meter (ML 50, A&D Company, Limited, Toshima, Japan).

#### 2.3.2. The Protease Activity of FP

The protease activity of FP was determined using the methods of Secades and Guijarro [18] and Oguntoyinbo et al. [19]. In brief, the crude enzyme solution obtained from the reaction sample was subjected to centrifugation at 15,000× *g* for 10 min at 4 °C. Subsequently, 100 μL of the supernatant was combined with 100 μL of 0.72 N NaOH (Macron Fine Chemicals™, Center Valley, PA, USA), and the absorbance at a wavelength of 420 nm was recorded to determine the enzyme activity unit (U/g). This unit was defined as the change in absorbance value over time following the interaction of the sample with the substrate.

#### 2.3.3. The Surfactin Analysis of FP

The samples were mixed with methanol (1:10, *w*/*v*), and the mixture was centrifuged at 10,000× *g* for 10 min at 4 °C to remove insoluble matter. Subsequently, the supernatant was filtered with a 0.22 μm polyvinylidene fluoride membrane to obtain a filtrate for surfactin analysis. The qualification and quantification of surfactin in the FP followed the procedure outlined by Lee et al. [4]. Analysis was conducted using liquid chromatography electrospray ionization high-resolution mass spectrometry (LC-ESI-HRMS, Thermo Fisher Scientific, Bremen, Germany). Full-scan LC-ESI-HRMS in positive ion mode showed a mass-to-charge ratio (*m*/*z*) ranging from 988 to 1200. The sample was injected at a volume of 5 μL into a reversed-phase column (Mightysil RP-18 GP, 250 × 4.6 mm; 5 μm, Kanto Kagaku, Okayama, Japan) for separation at a flow rate of 1 mL/min over a duration of 0 to 25 min. The mobile phase comprised 90% acetonitrile (Anaqua™ Chemicals Supply, Wilmington, DE, USA) and 10% water (*v*/*v*) with 0.1% formic acid (Merck, Hessen, Germany). The *m*/*z* of the surfactin standard (Sigma-Aldrich^®^, St. Louis, MO, USA) served as the basis for qualitative analysis. Meanwhile, a concentration gradient solution of the surfactin standard was prepared and analyzed under the aforementioned chromatographic conditions in order to establish a standard curve. Subsequently, the surfactin concentration in the FP was determined based on this standard curve.

#### 2.3.4. Feed Composition Analysis

Proximate feed analysis was conducted according to the procedure of AOAC [20] to analyze the moisture (method 930.15), crude protein (method 984.13), ether extract (method 920.39), ash (method 942.05), calcium (method 968.08), and phosphorus (method 965.17) contents. The LYS1 count, protease activity, and surfactin content in the feed were analyzed as described above.

#### 2.3.5. Growth Performance

The BW, feed intake (FI), and survival rate (SR) were recorded at wk 0, 3, and 5. Weight gain (WG), feed conversion ratio (FCR), and production efficiency factor ([PEF] = [SR {%} × BW {kg}]/[age {day} × FCR] × 100) were calculated throughout the experiment.

#### 2.3.6. Sample Collection

Twenty-four broilers formed each group, with equal numbers of both sexes, and were euthanized for sample collection following a withdrawal period from feed for 12 h at 5 weeks old. Carcass traits, intestinal morphology, tibial bone characteristics, and clinical blood biochemistry were analyzed in all groups. Each analysis was performed in six replicates (n = 6), with four broilers included in each replicate.

#### 2.3.7. Carcass Traits

Various organs and tissues, including the heart, liver, gallbladder, proventriculus, gizzard, spleen, intestine (from the duodenum to rectum), abdominal fat (from the gizzard to celiac fat), breast (with bone and skin removed), and thigh (from the femur to the tibia, with bone and skin removed), were assessed for weight, and their relative weight in relation to body weight was determined.

#### 2.3.8. Intestinal Morphology

Three-centimeter sections of jejunum located before Meckel’s diverticulum and ileum situated after Meckel’s diverticulum were procured and preserved in 10% buffered formaldehyde for 48 h. Subsequently, each segment was embedded in paraffin, and a 5 μm slice of each specimen was obtained using a microtome, mounted on a glass slide, and stained with hematoxylin and eosin. The histological sections of the intestinal tissue were analyzed utilizing an optical microscope (Labophot-2, Nikon, Tokyo, Japan). Morphometric parameters were assessed using the ImageJ software (version 1.52v) (http://rsb.info.nih.gov/ij/, accessed on 4 May 2020). Measurements of the villus height, villus width, crypt depth, and tunica muscularis thickness of the small intestine were conducted at 10 different points for each parameter, and the villus height/crypt depth (V/C) ratio was computed. Villus height was determined from the apex of the crypt to the lamina propria of the villus. Villus width was measured from one side to the other at the midpoint of the villus. Crypt depth was defined as the shortest vertical distance from the point of contact with the villus to the mucous membrane. The thickness of the tunica muscularis was calculated as the distance between the lamina muscularis mucosae internally and the tunica serosa externally. The measurement of villus surface area was performed using the method of Liu et al. [21]. In brief, the surface area of the villi = π·w·h (where w represents villus width and h represents villus height).

#### 2.3.9. Tibial Bone Characteristics

The measurement of tibial bone characteristics was performed using the method of Mutuş et al. [22]. The labeled drumsticks were boiled at 100 °C for 10 min, then cooled to room temperature and manually de-fleshed, with removal of soft tissues and the patella. Then, the tibiotarsal length and bone weight were determined after air-drying for 24 h at room temperature. Before breaking, each bone was marked at its midpoint, and external diameters were measured perpendicular and parallel to the direction of applied force using a caliper. After breaking, diameter measurements were taken inside and outside the mid-shaft of the bone, perpendicular and parallel to the direction of applied force, in order to calculate the area moment of inertia. This parameter was used with elastic deformation to calculate the stress (kg/cm^2^). The thickness of the medial and lateral walls was measured at the midpoint mark using a dial caliper. The diameter of the medullary canal was determined by subtracting the thicknesses of the medial and lateral walls from the diameter at the diaphysis. The tibiotarsus weight/length, tibiotarsal, and robusticity indexes were calculated by the following formulae: (a) tibiotarsus weight/length index = tibia weight/tibial length; (b) tibiotarsal index = diaphysis diameter − medullary canal diameter/diaphysis diameter × 100; (c) robusticity index = bone length/cube root of bone weight. To analyze ash, Ca, and P contents in the tibia, bones were oven-dried at 105 °C for 24 h and were ashed in a muffle furnace at 600 °C for 6 h. The contents of ash (method 942.05), Ca (method 968.08), and P (method 965.17) in the tibia were analyzed as described by AOAC [20].

#### 2.3.10. Clinical Blood Biochemistry

Blood samples were taken from the brachial vein of broilers, followed by the collection of serum after centrifugation of the blood samples at 1734× *g* for 15 min. The serum was then preserved at −20 °C for subsequent analysis. The blood biochemistry of the serum was assessed, encompassing the activities of enzymes, such as aspartate aminotransferase (AST), lactate dehydrogenase (LDH), creatine kinase (CK), alkaline phosphatase (ALKP), and the levels of various components, including total protein (TP), albumin (ALB), blood urea nitrogen (BUN), uric acid (UA), total cholesterol (TC), triacylglyceride (TG), calcium (Ca), and phosphate (P). This analysis was conducted using an automated blood chemical analyzer equipped with Roche testing kits (Roche Cobas Mira Plus, Switzerland). Additionally, the albumin-to-globulin (A/G) ratio was calculated. The enzymatic activities and concentrations of the analyzed parameters were determined spectrophotometrically according to the methodology outlined by Akiba et al. [23].

### 2.4. Statistical Analysis

The continuous variables in the physiochemical characterizations, protease activity and surfactin content of FP, growth performance, carcass traits, intestinal morphology, tibial bone characteristics, and clinical blood biochemistry were analyzed according to the following statistical model:

*Y_ij_* = *μ* + *τ_i_* + *β_j_* + *e_ij_* (1)

where *Y_ij_* represents the measured value on the *i*-th treatment in the *j*-th block, *μ* is the overall mean, *τ_i_* is the fixed effect of the *i*-th treatment, *β_j_* is the random effect of the *j*-th block, and *e_ij_* is the random error associated with *Y_ij_*. The data were analyzed using the MIXED procedure (SAS 9.4), the groups were compared using a one-way ANOVA with a Tukey post hoc test, and coefficients for linear and quadratic contrasts were determined using the IML procedure (SAS 9.4) for the effect of LYS1 FP supplementation, with *p* < 0.05 indicating a statistically significant difference. The survival rate of growth performance was analyzed using the NPAR1WAY procedure (SAS 9.4). The groups were compared using SAS^®^ macro implementation of a multiple comparisons test as described by Elliott and Hynan [24], with *p* < 0.05 indicating a statistically significant difference.

The BLQ ascending model was fitted to growth performance, intestinal villi morphology, and tibial bone characteristics to further estimate the optimal LYS1 FP supplementation, and the results were analyzed according to the following statistical model:

*Y_ij_ = φ* + *β*_1_ × (*ω* − *X_i_*) + *β*_2_ × (*ω* − *X_i_*)^2^ + *b_j_* + *e_ij_* for *X_i_* < *ω*, and *Y_ij_* = *φ*+ *b_j_* + *e_ij_* for *X_i_* ≥ *ω* (2)

where *Y_ij_* represents the observed value associated with the pen randomly assigned to the FP supplementation *i* within block *j*, *φ* is the unknown maximum response (plateau) under the BLQ model, *β*_1_ and *β*_2_ are the corresponding unknown regression coefficients describing the relationship between *X_i_* and *Y_ij_* for values of *X_i_* smaller than the plateau, *ω* is the unknown minimum level of FP supplementation to reach the plateau under the BLQ model, *X_i_* is the *i*-th known FP supplementation, *β_j_* is the random effect of the *j*-th block, and *e_ij_* is the random error associated with the experimental unit in the *j*-th block that received the *i*-th FP supplementation. The data were analyzed using the NLMIXED procedure (SAS 9.4).

## 3. Results

### 3.1. The Physiochemical Characterizations and Functional Components of LYS1 FP

Table 2 shows the physiochemical characterizations of LYS1 FP. The LYS1 count and pH value increased to 9.36 log CFU/g and pH 7.35 (*p* < 0.05), respectively, after one or two days of fermentation, and then decreased (*p* < 0.05) after the drying and grinding treatment. The LYS1 count, protease activity, and surfactin content of LYS1 FP were 9.10 log CFU/g, 305 U/g, and 11.23 mg/g, respectively.

### 3.2. The Surfactin Composition of LYS1 FP

**Figure 1:**
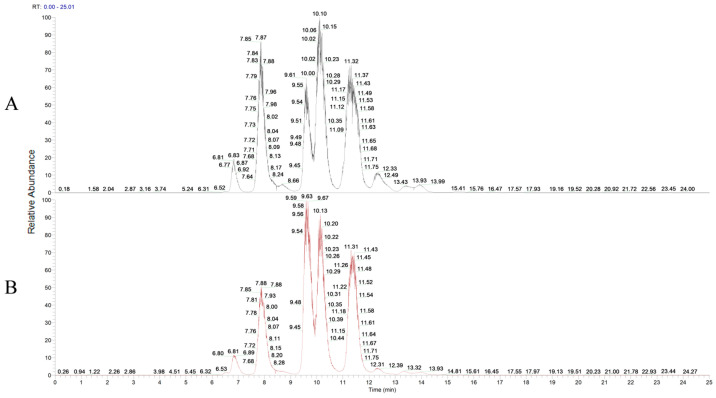
Chromatograms of surfactin from LYS1 FP ^1^: (**A**) surfactin standard; (**B**) LYS1 FP. ^1^ LYS1 FP: fer-mented product produced by Bacillus subtilis LYS1.

**Figure 2:**
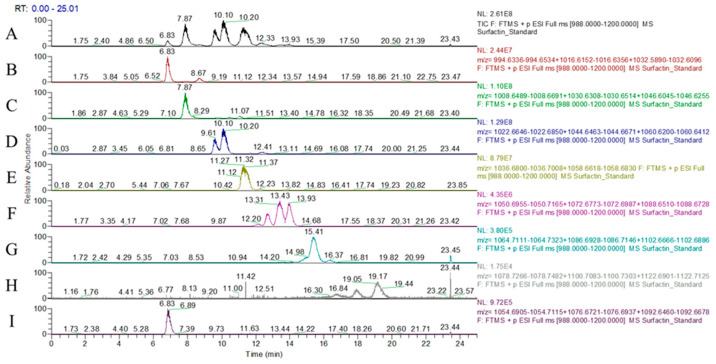
Chromatograms of different isoforms in surfactin standards: (**A**) total ion chromatogram; (**B**) C_12_ surfactin; (**C**) C_13_ surfactin; (**D**) C_14_ surfactin; (**E**) C_15_ surfactin; (**F**) C_16_ surfactin; (**G**) C_17_ surfactin; (**H**) C_18_ surfactin; (**I**) C_15_ linear surfactin.

Figure 1 shows the chromatograms of surfactin from LYS1 FP. The relative surfactin peaks were detected in LYS1 FP, but the peaks’ heights were not exactly the same as the standard. Figure 2 shows the different isoforms in the surfactin standard. The C_12_ to C_18_ surfactins were detected in the standard. In addition, the C_15_ surfactin contained linear and circular structures. The proton adducts [M+H] ^+^ *m*/*z* of the surfactins were C_12_: 994.64, C_13_: 1008.66, C_14_: 1022.67, C_15_: 1036.69, C_16_: 1050.71, C_17_: 1064.72, C_18_: 1078.74, and linear-C_15_: 1054.70. In addition, it was found that LYS1 FP contains the sodium and potassium adducts of surfactins. The [M+Na] ^+^ *m*/*z* of the surfactins were C_12_: 1016.63, C_13_: 1030.64, C_14_: 1044.64, C_15_: 1058.67, C_16_: 1072.69, C_17_: 1086.71, C_18_: 1100.72, and linear-C_15_: 1076.68. The [M+K] ^+^ *m*/*z* of the surfactins were C_12_: 1032.60, C_13_: 1046.61, C_14_: 1060.63, C_15_: 1074.64, C_16_: 1088.66, C_17_: 1102.68, C_18_: 1116.69, and linear-C_15_: 1092.66. Table 3 shows the surfactin composition of LYS1 FP. The composition ratios of the LYS1 surfactins were C_12_: 3.81%, C_13_: 16.63%, C_14_: 52.56%, C_15_: 25.09%, C_16_: 1.59%, C_17_: 0.06%, C_18_: 0.02%, and linear-C_15_: 0.25%.

### 3.3. Growth Performance

Table 4 shows the effects of LYS1 FP supplementation on growth performance in broilers. At 0 to 3 wk, the FI of the 1.5% FP, 2.5% FP, and fish meal groups was higher than that of the 0% group (*p* < 0.05), and the WG of the 2% FP, 2.5% FP, and fish meal groups was higher than that of the 0% group (*p* < 0.05). In addition, only the 2% FP group showed an improved PEF (*p* < 0.05) compared with the 0% group. At 3 to 5 wk, the FI of the FP-supplemented and fish meal groups was higher than that of the 0% group (*p* < 0.05), and only the FP-supplemented groups showed an improved WG (*p* < 0.05) compared with the 0% group. At 0 to 5 wk, the FI and WG of the FP-supplemented and fish meal groups were higher than those of the 0% group (*p* < 0.05), and the FP-supplemented groups showed an improved PEF (*p* < 0.05) compared with the 0% group. The FI, WG, and PEF at 0 to 3, 3 to 5, and 0 to 5 weeks old showed a linear effect with increased FP supplementation (*p* < 0.05), and the FI and WG at 3 to 5 weeks old, BW at 5 weeks old, and WG at 0 to 5 weeks old showed a quadratic effect with increased FP supplementation (*p* < 0.05).

### 3.4. Carcass Traits

Table S1 shows the effects of LYS1 FP supplementation on carcass traits in broilers. The relative weight of the carcass, heart, liver and gallbladder, gizzard and proventriculus, spleen, intestine, abdominal fat, skinless breast, and whole legs showed no significant differences between groups (*p* > 0.05).

### 3.5. Intestinal Morphology

Table 5 shows the effects of LYS1 FP supplementation on intestinal morphology in broilers. In the jejunum, the villus height and V/C of the FP-supplemented and fish meal groups were higher than those of the 0% group (*p* < 0.05). In addition, the 2% FP group showed an improved surface area of villus (*p* < 0.05) compared with the 0% group. The villus height, crypt depth, villus width, V/C, and surface area of villus in the jejunum showed a linear effect with increased FP supplementation (*p* < 0.05), and the villus height and V/C showed a quadratic effect with increased FP supplementation (*p* < 0.05). In the ileum, the V/C of the FP-supplemented and fish meal groups was higher than that of the 0% group (*p* < 0.05), and only the 2% FP group showed an improved V/C (*p* < 0.05) compared with the fish meal group. In addition, the FP-supplemented and fish meal groups showed an improved crypt depth (*p* < 0.05) compared with the 0% group. The villus height, crypt depth, V/C, and surface area of villus in the ileum showed a linear effect with increased FP supplementation (*p* < 0.05), and the crypt depth and V/C showed a quadratic effect with increased FP supplementation (*p* < 0.05).

### 3.6. Morphometric Parameters, Bone Strength Measurements, and Mineral Contents of the Tibia

Table 6 shows the effects of LYS1 FP supplementation on the morphometric parameters, bone strength measurements, and mineral contents of the tibia in broilers. The force of the 2% FP, 2.5% FP, and fish meal groups was higher than that of the 0% group (*p* < 0.05). In addition, only the 2.5% FP group showed an improved tibiotarsal index (*p* < 0.05) compared with the 0% group. The force, tibiotarsal index, Ca, and P in the tibia showed a linear effect with increased FP supplementation (*p* < 0.05). Moreover, the tibial weight and tibiotarsus weight/length index showed a quadratic effect with increased FP supplementation (*p* < 0.05).

### 3.7. Clinical Blood Biochemistry

Table 7 shows the effects of LYS1 FP supplementation on clinical blood biochemistry in broilers. The AST and LDH activities of the 2% FP group were higher than those of the 0% group (*p* < 0.05), and the CK activity of the 0% group was higher than that of the 1.5–2.5% FP and fish meal groups (*p* < 0.05). The A/G of the 2.5% FP group was higher than that of the 2% FP group (*p* < 0.05). The activities of AST, LDH, and CK, as well as the contents of TC and TG showed a linear effect with increased FP supplementation (*p* < 0.05).

### 3.8. The optimal Supplementation of LYS1 FP for Growth Performance, Intestinal Morphology, and Tibial Bone Characteristics

Table 8 shows the optimal supplementation of LYS1 FP for growth performance, intestinal morphology, and tibial bone characteristics in broilers. The fitted broken-line quadratic models of the optimal supplementation of LYS1 FP for growth performance, intestinal morphology, and tibial bone characteristics in broilers are displayed in Table S2. In terms of growth performance, the optimal supplementation of LYS1 FP for BW at 5 weeks old and WG at 3–5 and 0–5 weeks old was 1.8%, and the optimal supplementation for FI at 3–5 weeks old was 0.9%. In terms of intestinal morphology, the optimal supplementation of LYS1 FP for villus height in the jejunum and crypt depth in the ileum was 1.8 and 1.7%, respectively. The optimal supplementation for V/C in the jejunum and ileum was 1.9%. In terms of tibial bone characteristics, the optimal supplementation of LYS1 FP for tibial weight was 1.5%, and the optimal supplementation for tibiotarsus weight/length index was 1.6%.

## 4. Discussion

The variations in *Bacillus*-like counts and pH values are often used as indicators to evaluate fermentation quality. The counts of the *Bacillus* strain increased during fermentation, indicating that the strain was suitable for growth under fermentation conditions. The water content of the substrate decreased during the drying and grinding processes, which resulted in decreases in overall water activity and *Bacillus*-like counts. Nevertheless, *Bacillus* endospores can resist high temperatures and dry conditions during processing [2,25]. The pH value increases during fermentation because the *Bacillus* strain degrades the protein of the substrate to produce amines and ammonia, and the high temperature of the drying and grinding processes causes the ammonia to evaporate with the water, reducing the pH of the FP [4,14]. The surfactin yield of the FP is positively related to the density of the production strain [26]. In a previous study, LYS1 was selected as a strain that could produce high surfactin yields. However, the water content of the substrate dropped to 33% when fermented for 2 days, reducing the water activity and affecting the fermentation results of LYS1 [4]. In this study, the improved ventilation equipment used in the fermentation process reduced the water evaporation so that the water content of the substrate was maintained at 50–55% during fermentation. Therefore, the LYS1 counts increased by 608% (9.1 vs. 8.25 log CFU/g FP), which indirectly increased the surfactin content of the FP by 5.05% (11.23 vs. 10.69 mg/g FP) compared with the previous study.

The type and proportion of adducts affect the quantitative and qualitative results of surfactins [27,28]. This study used the positive ion mode in LC-ESI-HRMS to improve the analysis method and was able to detect the C_12_ to C_18_ surfactin isoforms in LYS1 FP. Moreover, the sodium and potassium adducts were detected in LYS1 FP, except for the proton adducts. The composition types and ratios of surfactin produced by *B. subtilis* differ according to the strain characteristics and fermented conditions, and the bioactivity of surfactin increases with the extension of the β-hydroxy fatty acid chains [29,30,31]. Dhanarajan et al. [6] indicated that the minimum inhibitory concentration of C_16_ surfactin for *Escherichia coli* was 3.75 μg/mL, which was much lower than that of C_13_ surfactin (>120 μg/mL). Compared with the study of Lee et al. [4], this study found reduced water evaporation during fermentation. Consequently, this increased the content of LYS1 surfactin and affected the surfactin composition, and a new long-carbon-chain C_18_ surfactin was found.

Supplementation with 1 to 2.5% LYS1 FP showed better WG, FI, and PEF at 0 to 5 weeks old (*p* < 0.05), which was similar to the results of Lee et al. [4]; supplementation with 2.5% LYS1 FP could replace the same proportion of fish meal in the broilers’ diet, and the WG and PEF of the LYS1 group were better than those of the unfermented group. Thus, this study again confirmed the improved effects of LYS1 FP on the growth performance of broilers, which was effectively improved by just supplementation with 1% LYS1 FP, and the improvement showed a linear effect with increased FP supplementation (*p* < 0.05). In addition, the FI and WG at 3 to 5 weeks old and the WG at 0 to 5 weeks old showed a quadratic effect with increased FP supplementation (*p* < 0.05), indicating that an optimal supplementation with LYS1 FP for FI and WG in broilers exists. *B. subtilis* is generally used as a direct-fed microbial applied in diets to improve growth and intestinal health in poultry production [2,32,33]. Feed substrates fermented by *B. subtilis* can transform macromolecular nutrients into more digestible micromolecular ones and reduce the antinutritional factors to improve the utilization of nutrients by broilers [34,35]. *B. subtilis* can produce functional components such as extracellular enzymes (e.g., protease) and antibacterial peptides (e.g., surfactin) during fermentation, which can improve the intestinal health of broiler chickens during their early growth period [2,36]. The authors’ previous study confirmed that the *Bacillus*-like counts, protease activity, and surfactin content of FP were moderately correlated with the growth performance of broilers [4]. The newly discovered long-carbon-chain C_18_ surfactin can increase the antibacterial activity of LYS1 FP in broilers’ intestines. Therefore, *B. subtilis* FP not only improves the utilization of substrate nutrients but also provides the benefits of probiotics and secondary metabolites, which promote broilers’ intestinal morphology and growth performance [12,14].

*B. subtilis* can produce particular flavors according to different fermented conditions, influencing the palatability of the FP to poultry [37,38,39]. Fish meal contains rich amino acid compositions and easily digestible proteins, is often used for high-quality protein in poultry diets, and affects palatability according to its quality [14,40]. This study used the high-yield surfactin strain LYS1 to produce the FP, and the fish meal was a high-quality brand (TripleNine Group) with CP 70%. The FP-supplemented and fish meal groups showed an increased FI at different growth periods (*p* < 0.05), showing that the LYS1 FP and fish meal had better palatability to the broilers, thereby improving their WG. A higher PEF value means better economic profits in poultry production [41,42]. The content and composition of surfactin and the LYS1 counts of LYS1 FP were better than the results of Lee et al. [4]. At 0 to 5 wk, the WG of the FP-supplemented groups was better than that of the 0% group (*p* < 0.05), which could achieve the same performance as the fish meal group. Consequently, supplementation with 1 to 2.5% LYS1 FP improved the PEF at 0 to 5 weeks old (*p* < 0.05), compared with the 0% group. This shows that LYS1 FP has excellent potential for use in the production of broilers throughout their entire growth period.

The authors’ previous study indicated that supplementation with 2.5% LYS1 FP or fish meal did not affect the relative weight of the carcass, organs, and tissues in broilers [4]. Lee et al. [14] indicated that supplementation with 5% FP produced by *B. amyloliquefaciens* CU33 did not affect the relative weight of the carcass, heart, liver, abdominal fat, skinless breast, and whole legs in broilers. These findings are similar to the results of this study, which showed that supplementation with 1 to 2.5% LYS1 FP did not show any negative effects on broilers’ carcass traits.

The V/C is generally an indicator of intestinal digestibility [43,44]. A higher villus height means a larger intestinal surface area and mature epithelial cells, improving nutrient absorption. Furthermore, a shallower crypt depth means reduced epithelial cell proliferation and reduced energy consumption. The surface area of villus is the curved area of villus height multiplied by villus width and π; a higher value means a more extensive contact surface area between villi and nutrients. Thus, the higher the V/C and surface area of villus, the better the digestibility and absorption of nutrients in the small intestine. Most studies indicate that supplementation with *B. subtilis* or its FP can improve the villus height, crypt depth, villus width, V/C, and surface area of villus in the small intestine [11,45,46]. The authors’ previous study found that supplementation with 2.5% LYS1 FP improved villus height, crypt depth, and V/C in the jejunum compared with an unfermented group [4]. In this study, supplementation with 1 to 2.5% LYS1 FP improved the villus height, crypt depth, V/C, and surface area of villus (*p* < 0.05) compared with the 0% group. This again confirms the improved effects of LYS1 FP on intestinal villus development, thereby improving the growth performance of broilers (Table 4).

The digestion and absorption of starch, protein, and fat in broilers mainly occur in the jejunum, while the digestion and absorption of some residual nutrients may occur in the ileum, but the primary function of this intestinal segment is to absorb water and minerals [47,48]. Supplementation with 1 to 2.5% LYS1 FP or 2.5% fish meal improved the V/C in the jejunum and ileum (*p* < 0.05), and supplementation with LYS1 FP could improve the surface area of villus in the jejunum (*p* < 0.05). The villus height, crypt depth, V/C, and surface area of villus in the jejunum and ileum showed a linear effect with increased FP supplementation (*p* < 0.05), and the villus height in the jejunum, crypt depth in the ileum, and V/C in both the jejunum and ileum showed a quadratic effect with increased FP supplementation (*p* < 0.05). This indicates that an optimal supplementation of LYS1 FP exists for villus height in the jejunum, crypt depth in the ileum, and V/C in the jejunum and ileum in broilers.

The higher the tibiotarsus weight/length index, the higher the density of the tibia. A higher tibiotarsal index represents better mineralization of the tibia [3,21]. In this study, supplementation with 1 to 2.5% LYS1 FP could linearly improve the force and tibiotarsal index in the tibia (*p* < 0.05), compared with the 0% group. Moreover, the tibiotarsus weight/length index showed a quadratic effect with increased FP supplementation (*p* < 0.05). These results indicate that LYS1 FP improved the mineralization and bone strength in the tibia, helping to support the heavier BW of broilers (Table 4). Ca and P are essential elements that constitute hydroxyapatite, the main component of bone, and are related to compressive strength [49]. Most studies indicate that adding *B*. *subtilis* to broilers’ diets can improve the strength, length, weight, index, and mineral composition (Ca and P) of the tibia [3,15,50]. Similarly, the FP-supplemented groups showed a linear increase in Ca and P contents in the tibia (*p* < 0.05), showing that LYS1 FP improved the accumulation capacity of Ca and P in bones. Supplementation with *B*. *subtilis* improved the mineralization and bone strength in the tibia, related to the improvements in the intestinal digestion of nutrients and absorption of Ca and P [3,51]. This study found that supplementation with LYS1 FP could improve the V/C in the ileum (*p* < 0.05), increasing the absorption of Ca and P in the ileum to improve tibial mineralization. Surfactin is an antibacterial lipopeptide produced by *B*. *subtilis*; its heptapeptide ring contains two acidic amino acids (Glu _1_ and Asp _5_), forming a “claw”-like structure. The pKa of these two amino acid residues is 5.4, above which they will dissociate; therefore, this structure is a potential binding site for divalent cations [52]. In the alkaline environment of the small intestine, in addition to its original antibacterial function, surfactin may also help broilers to absorb Ca and other minerals. This hypothesis needs further exploration to confirm it. This study found that adding FP produced by the high-yield surfactin strain LYS1 to broilers’ diet could improve the mineralization, density, stiffness, and composition (Ca and P) of the tibia, and the improvement showed a linear effect with increased FP supplementation (*p* < 0.05). This means LYS1 FP can improve the development of the tibia to support a faster increase in BW. The tibial weight and tibiotarsus weight/length index showed a quadratic effect with increased FP supplementation (*p* < 0.05), meaning that an optimal supplementation of LYS1 FP exists for improving tibial weight and tibiotarsus weight/length index in broilers.

Clinical blood biochemistry often uses blood enzymes to detect liver and muscle damage in animals. AST and LDH are widely distributed in broilers’ various tissues, and injury to the liver and skeletal muscle will increase AST and LDH activity. In addition, CK is a specific indicator of muscle cell damage [53]. Among these enzymes, the half-life order is AST > CK > LDH. When LDH activity increases, but CK activity does not increase in serum, there may be no muscle damage [54]. However, it should be noted that blood enzyme profiles can only be used as a rough indicator to explain elevated blood enzyme activity, and they do not represent the condition of a specific organ [55]. The authors’ previous study indicated that supplementation with 2.5% LYS1 FP did not affect the enzyme activity in clinical blood biochemistry in broilers [4]; this is not consistent with the results of this study. When the enzymes enter blood circulation, due to the different removal rates of various components, the composition of the enzyme profile may be changed and is affected by feed ingredients, nutritional status, season, gender, age, feeding, and management. The activities of AST and CK increase in commercial broilers due to the rapid growth rate of skeletal muscle mass [56]. Generally, when muscles are injured, CK activity increases first, followed by AST activity. When CK activity decreases and AST activity increases, it may indicate a muscle healing process. In addition, if muscle cells are dystrophic, their CK activity may also be increased [57]. LDH activity can be used to evaluate muscle metabolism, and AST activity may be affected by the body’s protein metabolism rate [58]. In this study, supplementation with 2% LYS1 FP showed higher WG (Table 4), meaning that the broilers were in a rapid growth period. Therefore, the 2% FP group showed higher activity of AST and LDH than the 0% group (*p* < 0.05), while the CK activity of the 1.5–2.5% FP and fish meal groups was lower than that of the 0% group (*p* < 0.05). This may have been because the muscles were already in the repair process. In addition, the FI of the 0% group was lower than that of the other groups (*p* < 0.05), which may have affected the physiological nutritional status of their muscles.

The contents of TP, ALB, TC, and TG in the blood may be affected by the FI and the diet composition. Yaman et al. [59] indicated that the contents of TP and ALB in broilers were reduced after restricted feeding. In this study, the FI of the 0% group was lower than that of the other groups (*p* < 0.05); thus, the 0% group showed lower contents of TP and ALB. The A/G of the 2.5% FP group was higher than that of the 2.0% FP group (*p* < 0.05), but there was no difference between them and the 0% group (*p* > 0.05), indicating that the blood protein composition in each group was in a normal state. The UA is the primary end product of nitrogen metabolism in poultry, which is often used together with BUN to evaluate the renal function of poultry [55]. Rajman et al. [60] indicated that broilers had lower contents of TC and TG after restricted feeding, similar to the results of the present study. The activity of AST and LDH in broilers, along with their contents of TC and TG, increased linearly with increased FP supplementation (*p* < 0.05), while the activity of CK decreased linearly (*p* < 0.05). This is consistent with the BW and FI in broilers increasing linearly with increased FP supplementation (*p* < 0.05) (Table 4). In addition, surfactin is a biosurfactant with the property of emulsifying oil [61]. LYS1 FP had a higher surfactin content, which may be related to its linear effect on increasing the levels of TC and TG in serum (*p* < 0.05), but further exploration is needed in order to confirm this.

Recent studies have found that feeding broilers with FP containing surfactin can improve their growth performance. Cheng et al. [11] found that supplementation with 0.3% *B. subtilis* NIU068 FP (the endospore count was 3.6 × 10^6^ CFU/kg feed, and the surfactin content was approximately 0.3 mg/kg feed) could improve BW and intestinal villus morphology in broilers, compared with the control group. Chen and Yu [13] found that supplementation with 0.3% *B. licheniformis* FP (the *B. licheniformis* count was 9 × 10^12^ CFU/kg feed, and the surfactin content was approximately 14.1 mg/kg feed) could improve broilers’ WG and regulated their fecal microbial flora. The authors’ previous study indicated that supplementation with 2.5% LYS1 FP (the LYS1 count was 9.64 log CFU/kg feed, and the surfactin content was 276 mg/kg feed) could improve growth performance in broilers, and the surfactin content and *Bacillus*-like counts of the FP were moderately correlated with growth performance [4].

This study found that adding 1 to 2.5% LYS1 FP (the LYS1 count was 10.10–10.49 log CFU/kg feed, and the surfactin content was 112.30–280.75 mg/kg feed, Table 1) to broilers’ diets could improve WG, V/C in the jejunum and ileum, and tibial mineralization. The optimal supplementation of LYS1 FP for WG is 1.8%, consistent with the finding that adding more than 2% LYS1 FP leads to heavier WG. The optimal supplementation of LYS1 FP for FI is 0.9%, implying that adding more than 1% LYS1 FP can increase FI in broilers. The optimal supplementation of LYS1 FP for V/C in the jejunum and ileum is 1.9%. This is consistent with the finding that adding more than 2% LYS1 FP leads to a better V/C, which can reduce intestinal energy consumption and increase the absorption of nutrients, thereby improving growth performance, and 2% LYS1 FP is optimal. The optimal supplementation of LYS1 FP for the tibiotarsus weight/length index is 1.6%, implying that adding more than 2% LYS1 FP leads to better tibial density, thereby supporting rapid weight growth in broilers. Based on the above fitting results obtained by the BLQ model, the optimal supplementation of LYS1 FP for broilers’ diets was adjusted to 1.9% (LYS1 count of 10.37 log CFU/kg feed, and surfactin content of 213.37 mg/kg feed), which can simultaneously improve the WG, V/C in the jejunum and ileum, and tibial bone density in broilers.

## 5. Conclusions

The LYS1 counts, surfactin content, and surfactin composition of the FP were 9.1 log CFU/g, 11.23 mg/g, and C_12_ to C_18_, respectively. Supplementing 1 to 2.5% LYS1 FP to the diet of broilers can improve their growth performance, the development of intestinal villi, and tibial bone density/mineralization. Based on the fitting results obtained with the BLQ model, the optimal supplementation of LYS1 FP is 1.9% for WG, intestinal villi morphology, and tibia development in broilers.

## Figures and Tables

**Table 1:** Ingredient composition of the basal diets.

Items	0–3 wk.						3–5 wk.					
	Fish Meal	LYS1 FP, %					Fish Meal	LYS1 FP, %				
		0	1	1.5	2	2.5		0	1	1.5	2	2.5
Corn, grain	48.71	45.86	45.86	45.86	45.86	45.86	59.22	56.38	56.38	56.38	56.38	56.38
Full fat soybean, 37.5% CP	28.96	34.49	34.49	34.49	34.49	34.49	17.87	23.37	23.37	23.37	23.37	23.37
Soybean meal, 43% CP	13.16	12.76	11.76	11.26	10.76	10.26	13.93	13.56	12.56	12.06	11.56	11.06
Soybean oil	2.00	2.00	2.00	2.00	2.00	2.00	2.00	2.00	2.00	2.00	2.00	2.00
Feather meal, 85% CP	1.25	1.25	1.25	1.25	1.25	1.25	1.25	1.25	1.25	1.25	1.25	1.25
999 LT fish meal, 70% CP	2.50	-	-	-	-	-	2.50	-	-	-	-	-
LYS1 FP ^1^	-	-	1.00	1.50	2.00	2.50	-	-	1.00	1.50	2.00	2.50
Calcium phosphate, 21% P	1.30	1.39	1.39	1.39	1.39	1.39	1.19	1.28	1.28	1.28	1.28	1.28
Limestone, pulverized	1.58	1.65	1.65	1.65	1.65	1.65	1.44	1.50	1.50	1.50	1.50	1.50
Salt	0.3	0.3	0.3	0.3	0.3	0.3	0.3	0.3	0.3	0.3	0.3	0.3
DL-Methionine	0.1	0.14	0.14	0.14	0.14	0.14	0.17	0.19	0.19	0.19	0.19	0.19
L-Lysine HCl, 78%	-	0.03	0.03	0.03	0.03	0.03	0.01	0.05	0.05	0.05	0.05	0.05
Vitamin premix ^2^	0.03	0.03	0.03	0.03	0.03	0.03	0.03	0.03	0.03	0.03	0.03	0.03
Mineral premix ^3^	0.10	0.10	0.10	0.10	0.10	0.10	0.10	0.10	0.10	0.10	0.10	0.10
Total	100	100	100	100	100	100	100	100	100	100	100	100
Analyzed value												
DM, %	92.1	91.9	91.9	92.2	92.3	91.8	91.8	92.2	91.8	91.9	92.2	91.7
CP, %	23.1	23.3	23.1	23	23.1	23.2	20.2	20.3	20.2	20.3	20.2	20.1
EE, %	7.61	8.26	8.24	8.24	8.25	8.23	8.44	9.13	9.12	9.11	9.14	9.11
Ash, %	1.87	1.88	1.87	1.89	1.87	1.87	1.66	1.66	1.66	1.67	1.65	1.65
Ca, %	1.05	1.03	1.03	1.05	1.04	1.07	0.92	0.91	0.90	0.91	0.92	0.93
P, %	0.72	0.73	0.73	0.72	0.72	0.71	0.69	0.68	0.67	0.66	0.67	0.67
LYS1 counts, log CFU/g	-	-	7.07	7.24	7.36	7.46	-	-	7.10	7.26	7.39	7.49
Protease activity, U/g	-	-	3.06	4.56	5.98	7.58	-	-	3.04	4.79	6.20	7.63
Surfactin, mg/g	-	-	0.11	0.16	0.21	0.27	-	-	0.11	0.18	0.23	0.28

^1^ LYS1 FP: fermented product produced by *Bacillus subtilis* LYS1. ^2^ Vitamin premix supplied per kilogram of diet: vitamin A, 50,000,000 IU; vitamin D3, 10,000,000 IU; vitamin E, 75 g; vitamin K3, 20 g; vitamin B1, 10 g; vitamin B2, 30 g; vitamin B6, 20 g; Ca-pantothenate, 60 g; niacin, 200 g; biotin, 100 mg; folic acid, 5 g; vitamin B12, 100 mg. ^3^ Mineral premix supplied per kilogram of diet: Cu (CuSO_4_·5H_2_O, 25.45% Cu), 8 mg; Fe (FeSO_4_·7H_2_O, 20.09% Fe), 100 mg; Mn (MnSO_4_·H_2_O, 32.49% Mn), 100 mg; Zn (ZnO, 80.35% Zn), 75 mg; Se (NaSeO_3_, 45.56% Se), 0.30 mg.

**Table 2:** Physicochemical characterizations of LYS1 FP ^1^.

Fermented Stage ^2^	LYS1 FP ^3^
	LYS1 counts, log CFU/g
Initial, 0 d	8.17 ± 0.18 ^c^
Fermentation, 1 d	9.30 ± 0.17 ^a^
Fermentation, 2 d	9.36 ± 0.09 ^a^
Dried fermented powder	9.10 ± 0.24 ^b^
*p*-Value	<0.001
	pH value
Initial, 0 d	6.06 ± 0.01 ^c^
Fermentation, 1 d	6.70 ± 0.02 ^b^
Fermentation, 2 d	7.35 ± 0.01 ^a^
Dried fermented powder	6.69 ± 0.01 ^b^
*p*-Value	<0.001
	Protease activity, U/g
Dried fermented powder	305 ± 24
	Surfactin, mg/g
Dried fermented powder	11.23 ± 0.35

^a–c^ Means in the same column with different superscripts are significantly different (*p* < 0.05). ^1^ Data are the means ± SD of 3 batches of each fermented product (FP), n = 3. ^2^ The water content ranged from 55 to 50% during fermentation and was below 12% during the drying and grinding processes. ^3^ LYS1 FP: fermented product produced by *Bacillus subtilis* LYS1.

**Table 3:** Surfactin composition of LYS1 FP ^1^.

Surfactin Isoform	LYS1 FP ^2^
	Composition, %
C_12_	3.81 ± 0.10
C_13_	16.63 ± 0.16
C_14_	52.56 ± 0.04
C_15_	25.09 ± 0.01
C_16_	1.59 ± 0.03
C_17_	0.06 ± 0.001
C_18_	0.02 ± 0.001
Linear-C_15_	0.25 ± 0.01

^1^ Data are the means ± SD of 3 batches of each fermented product (FP), n = 3. ^2^ LYS1 FP: fermented product produced by *Bacillus subtilis* LYS1.

**Table 4:** Effects of LYS1 FP supplementation on growth performance in broilers ^1^.

Period, Weeks Old	Fish Meal	LYS1 FP ^2^, %					SEM	*p*-Value	Effects of LYS1 FP	
		0	1	1.5	2	2.5			Linear	Quadratic
	BW, g/bird									
0	43	43	43	43	43	43	1.2	1.000	1.000	1.000
3	892 ^a^	806 ^b^	850 ^ab^	885 ^ab^	899 ^a^	893 ^a^	23.9	0.016	<0.001	0.320
5	2213 ^a^	2040 ^b^	2245 ^a^	2262 ^a^	2310 ^a^	2292 ^a^	44.1	<0.001	<0.001	0.025
	FI, g/bird									
0–3	966 ^a^	867 ^b^	899 ^ab^	957 ^a^	927 ^ab^	958 ^a^	40.3	0.038	0.007	0.591
3–5	1985 ^a^	1760 ^b^	2068 ^a^	1999 ^a^	2006 ^a^	2063 ^a^	52.4	0.001	<0.001	0.020
0–5	2951 ^a^	2627 ^b^	2967 ^a^	2957 ^a^	2933 ^a^	3021 ^a^	85.6	0.002	<0.001	0.051
	WG, g/bird									
0–3	850 ^a^	763 ^b^	807 ^ab^	843 ^ab^	857 ^a^	850 ^a^	24.2	0.015	<0.001	0.316
3–5	1321 ^ab^	1234 ^b^	1395 ^a^	1377 ^a^	1411 ^a^	1400 ^a^	32.8	0.006	<0.001	0.043
0–5	2171 ^a^	1997 ^b^	2202 ^a^	2220 ^a^	2268 ^a^	2250 ^a^	44.3	<0.001	<0.001	0.025
	FCR, FI/WG									
0–3	1.14	1.13	1.11	1.14	1.08	1.13	0.025	0.295	0.457	0.663
3–5	1.50	1.43	1.48	1.45	1.42	1.47	0.029	0.259	0.549	0.648
0–5	1.36	1.31	1.35	1.33	1.29	1.34	0.021	0.124	0.742	0.710
	PEF, (BW (kg) × SR ^3^ (%)/(FCR × day of age) × 100									
0–3	375 ^ab^	339 ^b^	363 ^ab^	373 ^ab^	396 ^a^	379 ^ab^	10.9	0.029	0.002	0.291
0–5	466 ^ab^	445 ^b^	476 ^a^	485 ^a^	511 ^a^	488 ^a^	9.8	0.003	<0.001	0.093

Abbreviations: BW, body weight; FCR, feed conversion ratio; FI, feed intake; PEF, production efficiency factor; SR, survival rate; WG, weight gain. ^a, b^ Means in the same row with different superscripts are significantly different (*p* < 0.05). ^1^ Data are the means of 6 pens of broilers, each pen with 10 broilers (n = 6). ^2^ LYS1 FP: fermented product produced by *Bacillus subtilis* LYS1. ^3^ SR in each group was 100%.

**Table 5:** Effects of LYS1 FP supplementation on intestinal morphology in broilers ^1^.

Items	Fish Meal	LYS1 FP ^2^, %					SEM	*p*-Value	Effects of LYS1 FP	
		0	1	1.5	2	2.5			Linear	Quadratic
	Jejunum									
Villus height, μm	1526 ^a^	1386 ^b^	1525 ^a^	1567 ^a^	1624 ^a^	1524 ^a^	30.0	<0.001	<0.001	0.003
Crypt depth, μm	213	237	214	213	216	209	9.2	0.325	0.045	0.362
Villus width, μm	192	165	181	197	237	212	19.9	0.200	0.024	0.823
Villus height/ crypt depth	7.23 ^a^	5.89 ^b^	7.15 ^a^	7.42 ^a^	7.62 ^a^	7.31 ^a^	0.269	0.002	<0.001	0.014
Surface area of villus, mm^2^	0.91 ^ab^	0.71 ^b^	0.87 ^ab^	0.97 ^ab^	1.21 ^a^	1.02 ^ab^	0.100	0.041	0.004	0.460
Thickness of the tunica muscularis, μm	350	352	356	346	347	349	13.4	0.996	0.746	0.982
	Ileum									
Villus height, μm	1041	916	1050	1017	1102	1098	54.6	0.168	0.013	0.567
Crypt depth, μm	211 ^b^	279 ^a^	204 ^b^	193 ^b^	183 ^b^	185 ^b^	9.5	<0.001	<0.001	0.002
Villus width, μm	155	150	147	147	159	155	4.6	0.385	0.222	0.317
Villus height/ crypt depth	4.97 ^b^	3.31 ^c^	5.16 ^ab^	5.27 ^ab^	6.07 ^a^	5.95 ^ab^	0.251	<0.001	<0.001	0.018
Surface area of villus, mm^2^	0.51	0.43	0.49	0.47	0.54	0.53	0.030	0.085	0.006	0.979
Thickness of the tunica muscularis, μm	361	347	351	431	315	388	29.3	0.142	0.570	0.643

^a–c^ Means in the same row with different superscripts are significantly different (*p* < 0.05). ^1^ Data are the means of 6 pens of broilers (n = 6). ^2^ LYS1 FP: fermented product produced by *Bacillus subtilis* LYS1.

**Table 6:** Effects of LYS1 FP supplementation on morphometric parameters, bone strength measurements, and mineral contents of the tibia in broilers ^1^.

Items	Fish Meal	LYS1 FP ^2^, %					SEM	*p*-Value	Effects of LYS1 FP	
		0	1	1.5	2	2.5			Linear	Quadratic
Length, cm	8.78	8.69	8.81	9.01	8.91	8.90	0.107	0.330	0.097	0.291
Weight, g	5.57	5.28	5.87	5.89	5.93	5.66	0.163	0.073	0.055	0.016
Force, kg	35.95 ^a^	25.67 ^b^	31.48 ^ab^	32.18 ^ab^	36.44 ^a^	32.18 ^a^	1.848	0.005	0.003	0.080
Tibiotarsus weight/length index, g/cm	63.56	60.82	66.64	65.32	66.47	63.63	1.703	0.178	0.169	0.031
Tibiotarsal index	88.40 ^ab^	77.07 ^b^	84.24 ^b^	82.41 ^b^	85.40 ^ab^	95.97 ^a^	4.180	0.009	0.001	0.250
Robusticity index	4.95	5.00	4.89	5.00	4.93	4.99	0.060	0.697	0.993	0.340
Yield stress, kg/cm^2^	358	324	353	369	382	374	34.7	0.509	0.061	0.554
Ash, %	44.32	43.9	44.91	43.62	45.29	44.48	0.871	0.768	0.536	0.831
Ca, %	18.48	17.92	19.03	19.72	20.43	19.55	0.713	0.194	0.036	0.313
P, %	12.38	11.04	12.37	12.93	13.12	12.38	0.588	0.217	0.045	0.106

^a, b^ Means in the same row with different superscripts are significantly different (*p* < 0.05). ^1^ Data are the means of 6 pens of broilers (n = 6). ^2^ LYS1 FP: fermented product produced by *Bacillus subtilis* LYS1.

**Table 7:** Effects of LYS1 FP supplementation on clinical blood biochemistry in broilers ^1^.

Items	Fish Meal	LYS1 FP ^2^					SEM	*p*-Value	Effects of LYS1 FP	
		0	1	1.5	2	2.5			Linear	Quadratic
AST, U/L	400 ^ab^	236 ^b^	361 ^ab^	272 ^ab^	575 ^a^	335 ^ab^	66.5	0.018	0.043	0.362
LDH, U/L	2945 ^ab^	1842 ^b^	2473 ^ab^	1904 ^ab^	3628 ^a^	2706 ^ab^	334.1	0.008	0.010	0.869
CK, U/L	2481 ^b^	7310 ^a^	4724 ^ab^	4299 ^b^	3638 ^b^	4608 ^b^	922.4	0.029	0.019	0.110
ALKP, U/L	4339	6742	6993	6013	6265	5072	1049.7	0.376	0.228	0.499
TP, g/dL	2.43	1.98	2.08	2.15	2.27	2.17	0.129	0.240	0.173	0.673
ALB, g/dL	1.33	1.13	1.20	1.22	1.22	1.28	0.060	0.283	0.101	0.943
A/G	1.27 ^ab^	1.33 ^ab^	1.33 ^ab^	1.32 ^ab^	1.17 ^b^	1.48 ^a^	0.055	0.016	0.555	0.054
BUN, mg/dL	0.75	0.63	0.57	0.62	1.85	0.65	0.404	0.213	0.327	0.809
UA, mg/dL	5.15	4.15	5.33	3.13	4.35	5.60	0.760	0.077	0.334	0.263
TC, mg/dL	90.8	72.2	80.5	81.8	85.3	89.3	4.67	0.096	0.012	0.944
TG, mg/dL	22.3	12.7	17.5	19.3	18.3	19.5	2.35	0.095	0.031	0.327
Ca, mg/dL	3.77	3.72	3.27	3.32	3.85	3.67	0.292	0.588	0.770	0.223
P, mg/dL	3.92	3.45	3.78	3.72	3.95	3.70	0.187	0.313	0.141	0.227

Abbreviations: A/G, albumin/globulin; ALB, albumin; ALKP, alkaline phosphatase; AST, aspartate aminotransferase; BUN, blood urea nitrogen; Ca, calcium; CK, creatine kinase; LDH, lactate dehydrogenase; P, phosphate; TC, total cholesterol; TG, triacylglyceride; TP, total protein; UA, uric acid. ^a, b^ Means in the same row with different superscripts are significantly different (*p* < 0.05). ^1^ Data are the means of 6 pens of broilers (n = 6). ^2^ LYS1 FP: fermented product produced by *Bacillus subtilis* LYS1.

**Table 8:** The optimal supplementation of LYS1 FP for growth performance, intestinal morphology, and tibial bone characteristics in broilers ^1^.

Items	Optimal LYS1 FP Supplementation, %	Asymptotic 95% CI
Growth performance		
BW at 5 weeks old	1.8	1.38–2.29
FI at 3–5 weeks old	0.9	0.44–1.33
WG at 3–5 weeks old	1.8	1.19–2.32
WG at 0–5 weeks old	1.8	1.34–2.28
Intestinal morphology		
VH in jejunum	1.8	1.39–2.17
VH/CD in jejunum	1.9	0.08–2.96
CD in ileum	1.7	1.62–1.79
VH/CD in ileum	1.9	0.07–3.11
Tibial bone characteristics		
Bone weight	1.5	0.52–2.47
TBLI	1.6	0.093–2.31

Abbreviations: BW, body weight; CD, crypt depth; FI, feed intake; TBLI, tibiotarsus weight/length index; VH, villus height; VH/CD, villus height/crypt depth; WG, weight gain. ^1^ LYS1 FP: fermented product produced by *Bacillus subtilis* LYS1.

## Data Availability

The data presented in this study are available on request from the corresponding author.

## Supplementary Materials

The following supporting information can be downloaded at https://www.mdpi.com/article/10.3390/ani14142079/s1, Table S1: Effect of LYS1 FP supplementation on carcass traits in broilers.; Table S2: The optimal supplementation for LYS1 FP for growth performance, intestinal morphology, and tibial bone characteristics in broilers as estimated based on fitted broken-line quadratic models.
