# Supplementary material for: The Optimal Supplementation of Fermented Product Produced by Bacillus subtilis Strain LYS1 with High Surfactin Yield for Improving Growth Performance, Intestinal Villi Morphology, and Tibial Bone Strength in Broilers
Source: Animals (Basel). 2024 Jul 16;14(14):2079. doi: 10.3390/ani14142079 (PMC11273593; doi:10.3390/ani14142079)
Supplement: Supplementary file 1 [file animals-14-02079-s001.zip › animals-3075440-supplementary.pdf]

**Table S1**: Effect of LYS1 FP supplementation on carcass traits in broilers [1].

| Items | Fish meal | LYS1 FP [2], % | | | | | SEM | *p*-Value | Effects of LYS1 FP | |
|---|---|---|---|---|---|---|---|---|---|---|
| | | 0 | 1 | 1.5 | 2 | 2.5 | | | Linear | Quadratic |
| Relative weight, g/100g live weight | | | | | | | | | | |
| Carcass | 81.0 | 80.5 | 80.2 | 80.2 | 80.6 | 80.0 | 1.44 | 0.995 | 0.863 | 0.995 |
| Heart | 0.48 | 0.51 | 0.57 | 0.56 | 0.51 | 0.49 | 0.040 | 0.475 | 0.618 | 0.101 |
| Liver and gallbladder | 1.78 | 1.69 | 1.57 | 1.64 | 1.76 | 1.65 | 0.073 | 0.255 | 0.777 | 0.485 |
| Gizzard and Proventriculus | 2.46 | 2.88 | 2.62 | 2.55 | 2.52 | 2.44 | 0.238 | 0.801 | 0.181 | 0.763 |
| Spleen | 0.08 | 0.11 | 0.10 | 0.09 | 0.08 | 0.08 | 0.018 | 0.720 | 0.143 | 0.992 |
| Intestine | 3.81 | 3.94 | 3.69 | 3.74 | 3.60 | 3.50 | 0.175 | 0.530 | 0.072 | 0.975 |
| Abdominal fat | 1.09 | 0.93 | 0.99 | 1.15 | 0.76 | 0.94 | 0.15 | 0.550 | 0.752 | 0.503 |
| Skinless breast | 20.9 | 20.4 | 21.5 | 21.2 | 21.1 | 21.8 | 0.59 | 0.650 | 0.174 | 0.814 |
| Whole legs | 21.3 | 21.6 | 21.5 | 20.6 | 21.6 | 20.3 | 0.44 | 0.227 | 0.110 | 0.644 |

[1] Data are the means of 6 pens of broilers (n = 6). [2] LYS1 FP: fermented product produced by *Bacillus subtilis* LYS1.

**Table S2**: The optimal supplementation for LYS1 FP for growth performance, intestinal morphology, and tibial bone characteristics in broilers as estimated based on fitted broken-line quadratic models [1].

| Items | Broken-line quadratic predictive equation |
|---|---|
| Growth performance | |
| BW at 5 weeks old | BW = 2297.96 - 3072.67 × (0.01836 - LYS1 FP supplementation) - 593335 × (0.01836 - LYS1 FP supplementation)$^2$, if LYS1 FP supplementation < 1.8%, and BW = 2297.96, if LYS1 FP supplementation ≥ 1.8%. |
| FI at 3-5 weeks old | FI = 2033.92 - 15359 × (0.009297 - LYS1 FP supplementation) - 1515134 × (0.009297 - LYS1 FP supplementation)$^2$, if LYS1 FP supplementation < 0.9%, and FI = 2033.92, if LYS1 FP supplementation ≥ 0.9%. |
| WG at 3-5 weeks old | WG = 1402.85 - 1136.1 × (0.01759 - LYS1 FP supplementation) - 463849 × (0.01759 - LYS1 FP supplementation)$^2$, if LYS1 FP supplementation < 1.8%, and WG = 2033.92, if LYS1 FP supplementation ≥ 1.8%. |
| WG at 0-5 weeks old | WG = 2255.35 - 3216.97 × (0.01827 - LYS1 FP supplementation) - 592488 × (0.01827 - LYS1 FP supplementation)$^2$, if LYS1 FP supplementation < 1.8%, and WG = 2255.35, if LYS1 FP supplementation ≥ 1.8%. |
| Intestinal morphology | |
| VH in jejunum | VH = 1569.91 - 14.764 × (0.01777 - LYS1 FP supplementation) - 599991 × (0.01777 - LYS1 FP supplementation)$^2$, if LYS1 FP supplementation < 1.8%, and VH = 1.62, if LYS1 FP supplementation ≥ 1.8%. |
| VH/CD in jejunum | VH/CD = 7.62 - 43 × (0.019 - LYS1 FP supplementation) - 2320 × (0.019 - LYS1 FP supplementation)$^2$, if LYS1 FP supplementation < 1.9%, and VH/CD = 7.62, if LYS1 FP supplementation ≥ 1.9%. |
| CD in ileum | CD = 183.14 - 4789.01 × (0.017 - LYS1 FP supplementation) - 555 × (0.017 - LYS1 FP supplementation)$^2$, if LYS1 FP supplementation < 1.7%, and CD = 183.14, if LYS1 FP supplementation ≥ 1.7%. |
| VH/CD in ileum | VH/CD = 6.07 - 69 × (0.019 - LYS1 FP supplementation) - 4148 × (0.019 - LYS1 FP supplementation)$^2$, if LYS1 FP supplementation < 1.9%, and VH/CD = 6.07, if LYS1 FP supplementation ≥ 1.9%. |
| Tibial bone characteristics | |
| Bone weight | Bone weight = 5.8917 - 20 × (0.015 - LYS1 FP supplementation) - 1386 × (0.015 - LYS1 FP supplementation)$^2$, if LYS1 FP supplementation < 1.5%, and Bone weight = 5.89, if LYS1 FP supplementation ≥ 1.5%. |
| TBLI | TBLI = 66.3808 - 141 × (0.01635 - LYS1 FP supplementation) - 12884 × (0.01635 - LYS1 FP supplementation)$^2$, if LYS1 FP supplementation < 1.6%, and TBLI = 66.3808, if LYS1 FP supplementation ≥ 1.6%. |

Abbreviations: BW, body weight; CD, crypt depth; FI, feed intake; TBLI, tibiotarsus weight/length index; VH, villus height; VH/CD, villus height/crypt depth; WG, weight gain. [1] LYS1 FP: fermented product produced by *Bacillus subtilis* LYS1.
